# Model-based prediction of maximum pool size in the ribbon synapse

**DOI:** 10.1186/1471-2202-16-S1-P41

**Published:** 2015-12-18

**Authors:** Caitlyn M Parmelee, Matthew Van Hook, Wallace B Thoreson, Carina Curto

**Affiliations:** 1 Department of Mathematics, University of Nebraska-Lincoln, Lincoln, NE 68588, USA; 2 Department of Ophthalmology and Visual Sciences, University of Nebraska Medical Center, Omaha, NE 68198, USA; 3 Department of Pharmacology and Experimental Neuroscience, University of Nebraska Medical Center, Omaha, NE 68198, USA; 4 Department of Mathematics, The Pennsylvania State University, University Park, PA, 16802, USA

## 

**Figure 1:**
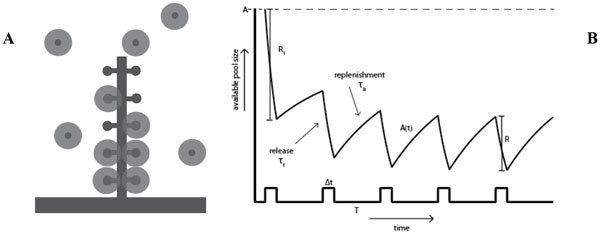
**A) The synaptic ribbon**. **(B) **The available pool size, A(t), during a stimulus pulse train.

The synaptic ribbon is a specialized structure in photoreceptor neurons that tethers vesicles prior to release (Figure 1A). When a cell is stimulated, vesicles are released from the ribbon and later replenished from the population of mobile vesicles in the synaptic terminal. A train of depolarizing pulses causes the ribbon to alternate between periods of release (lasting Δt = 25 ms) and replenishment (lasting T = 50ms), which occur on estimated timescales of τ_r _= 5 ms (for release) and τ_a _= 815 ms (for replenishment). After the first few pulses, the system approaches a limit cycle, and the amount of vesicles released on each pulse converges to a limiting value, R (Figure 1B). This can be used to determine the maximum available pool size on the ribbon, A. The standard method for estimating A is to measure the rate of replenishment in the limit, and then back-extrapolate from the cumulative release plot to obtain the available pool size at the start of the pulse train [1]. When comparing pulse trains of different strengths, this method yields substantially different values for A, a somewhat paradoxical result. Back-extrapolation assumes, however, that the replenishment rate is constant, even though it is thought to be proportional to the available space on the ribbon [2].

**Table 1:** Maximum pool size predictions from pulse train data

Stimulus	Estimate for A, from back-extrapolation	Estimate for A, from the model
-10 mV (stronger)	-136.8794 pA	-131.6858 pA
-30 mV (weaker)	-75.1020 pA	-133.6100 pA

We developed a model-based approach to estimate A from the limiting release R. We modeled the rate of release (resp. replenishment) to simply be proportional to the number of vesicles on the ribbon (resp. vacant ribbon sites), and using the measured timescale τ_r _(resp. τ_a_). By solving the alternating differential equations, we derived a recurrence relation for the release during each pulse, R_i_, which we then solved to obtain a closed form expression for R_i _and the limiting release R. Specifically, we found that A = cR, where c is a function of τ_r_,τ_a_,Δt,T, and p, with p a release constant that captures the stimulus dependence of release probabilities, and can be estimated from the first release, R_1_. In contrast to the back-extrapolation method, our model-based estimate for A was similar across stimulus types (Table 1), while p was much smaller for the weaker stimulus. This suggests that available pool size does not change with stimulus strength; instead, differences in release result from changes in release probability.
